# Distribution of deadwood and other forest structural indicators relevant for bird conservation in Natura 2000 special protection areas in Poland

**DOI:** 10.1038/s41598-021-94392-1

**Published:** 2021-07-22

**Authors:** Leszek Bujoczek, Małgorzata Bujoczek, Stanisław Zięba

**Affiliations:** 1 Department of Forest Resources Management, Faculty of Forestry, University of Agriculture in Krakow, Al. 29 Listopada 46, 31-425 Kraków, Poland; 2 Department of Forest Biodiversity, Faculty of Forestry, University of Agriculture in Krakow, Al. 29 Listopada 46, 31-425 Kraków, Poland

**Keywords:** Biodiversity, Forest ecology, Forestry

## Abstract

Numerous bird species, often rare or endangered, rely on the presence of standing and downed deadwood for shelter, nesting, and foraging. Habitat quality was evaluated on the basis of deadwood volume, the density of large standing deadwood, and the space filling index (SFI). The SFI reflects the degree of space filling of the bottom layers taking into account tree trunks, seedlings, saplings, ground vegetation, stumps, and downed deadwood. Analysis encompassed all special protection areas (SPAs) in Poland (a total of 107 SPAs containing 7974 sample plots monitored under the National Forest Inventory). An additional in-depth analysis was conducted for 30 SPAs with the greatest share of forest habitats. The studied indicators varied substantially both between and within individual SPAs, with deadwood volume ranging from 1.3 to 50.5 m^3^ ha^−1^ (mean of 9.0 m^3^ ha^−1^) and the density of large standing deadwood (diameter at breast height ≥ 30 cm) from 0.1 to 16.0 ind ha^−1^ (mean of 2.2 ind ha^−1^). These values were relatively low compared to the density of living trees with corresponding dimensions (111 ind ha^−1^). SFI analysis indicated high or very high space filling of the bottom forest layers on 14–56% of sample plots in a given SPA. The presence of deadwood was found to be significantly positively affected by SPA location in the mountains, a greater proportion of sites with higher fertility, a greater share of forest area under strict protection, as well as higher stand volume within a given SPA. The correlation between deadwood volume and the density of birds (primary and secondary cavity nesters) in individual SPAs was positive (*R* = 0.60). As compared to lowland areas, SPAs in mountain areas are generally characterized by high stand volumes, a greater density of large living trees, and a greater amount of diverse deadwood. In those areas conservation measures should involve continuous monitoring and diagnosing of any problems associated with the populations of individual bird species; focused efforts should be implemented to support those species that exhibit unfavorable population trends. In most lowland SPAs measures aimed at the improvement of site conditions for birds must be more extensive than in the mountains, with a low abundance of dead trees (especially large ones). These parameters can be improved by retaining some senescent stands in managed forests until their natural death and implementing a strict protection regime in areas of high conservation value.

## Introduction

The Natura 2000 network was created with a view to coordinating biodiversity conservation efforts across Europe^1^. As regards the conservation of avifauna, much of which consists of migratory species, Natura 2000 special protection areas (SPAs) were designated on the basis of the preexisting global network of Important Bird Areas (IBAs). The objective was to extend conservation efforts to all wild avian species under the 1979 Birds Directive^2^. It is thought that a third of them remain insufficiently protected^3^, with habitat degradation and loss being the most serious threats^4–6^.

Natura 2000 sites encompass multiple types of aquatic, waterside, heath and scrub, meadow, grassland, bog, rocky, and forest habitats^7^, whose quality is critical to supporting biodiversity. A considerable body of research has reported a positive correlation between habitat heterogeneity/diversity and animal species diversity. The higher the availability of diverse ecological niches, the greater the likelihood of the habitat attracting new species^8,9^. In particular, the complexity and biodiversity of forest ecosystems is closely associated with the presence of deadwood^10^ and tree-related microhabitats^11,12^. Of importance are also stand structure and species composition, the density of the various forest layers, and the presence of large dying and uprooted trees, and cavities^13–15^. Stand structure complexity is generally considered a good indicator of forest biodiversity^16^.

Different microhabitats and fine-scale habitat features may be important for different bird guilds or ecological groups^17,18^. For many bird species, habitat types associated with dying or standing dead trees offer unique foraging and nesting opportunities^19^. The species using standing deadwood include woodpeckers^20,21^ as well as numerous secondary cavity nesters, such as the nuthatch *Sitta europaea*, Eurasian pygmy owl *Glaucidium passerinum*, and red-breasted flycatcher *Ficedula parva*^22–24^. Of particular importance are large trees with a diameter at breast height (DBH) of at least 20–30 cm^25,26^, but cavities are often located in thicker trees. For instance, the mean DBH of trees with cavities of the great spotted woodpecker *Dendrocopos major*, black woodpecker *Dryocopus martius*, and European pied flycatcher *Ficedula hypoleuca* exceed 40 cm^27–29^. Also the bottom forest layer with natural microhabitats, such as fallen logs, upturned root plates, broken stumps, and patches of tall undergrowth plants provides foraging and nesting niches for numerous species^30,31^.

The presence of deadwood in forests depends on numerous factors, including forest type, site conditions, protection type, and terrain^32,33^. Both deadwood volume and diversity have an effect on bird density, species composition, and proportions between birds with different nesting preferences^9,34^. The relationship between microhabitats and bird assemblages has been analyzed in typical old-growth forests^23,35^, managed forests^36,37^, as well as plantations^34^. Research on retention forests has suggested that minimum retention levels ranging between 40 and 60% of the original habitat are needed in order to maintain the same bird assemblage as in the autochthonous unharvested forest, with particular regard to forest specialists^38^.

The literature also provides information about minimum desirable deadwood volumes with reference to various organisms, including birds^39,40^. A review of data from European forests has revealed thresholds ranging from 10 to 80 m^3^ ha^−1^ for boreal and lowland forests and from 10 to 150 m^3^ ha^−1^ for mixed montane forest, with the peak values being 20–30 m^3^ ha^−1^ for boreal forests, 30–40 m^3^ ha^−1^ for mixed montane forests, and 30–50 m^3^ ha^−1^ for lowland oak-beech forests^39^.

This study evaluated habitat quality in 30 special protection areas in Poland by determining the mean values of selected forest structural indicators, with a focus on the availability of deadwood, which is widely used in habitat quality analyses targeting bird populations. The application of surrogates or bioindicators represents shortcuts in ecology: a cost-effective strategy to study extremely complex systems^41,42^. Habitat quality was described based on a very large set of data collected from the National Forest Inventory. The paper is supplemented with an overview of bird densities in the analyzed SPAs based on literature data. The main research questions were as follows: (1) What is deadwood volume variability among SPAs?, (2) What are the characteristics and density of standing deadwood?, (3) To what extent is the bottom forest layer filled with deadwood, seedlings, saplings, and ground vegetation? and (4) What factors affect the availability of deadwood in SPAs?

## Methodology

### Study area and material

**Figure 1:**
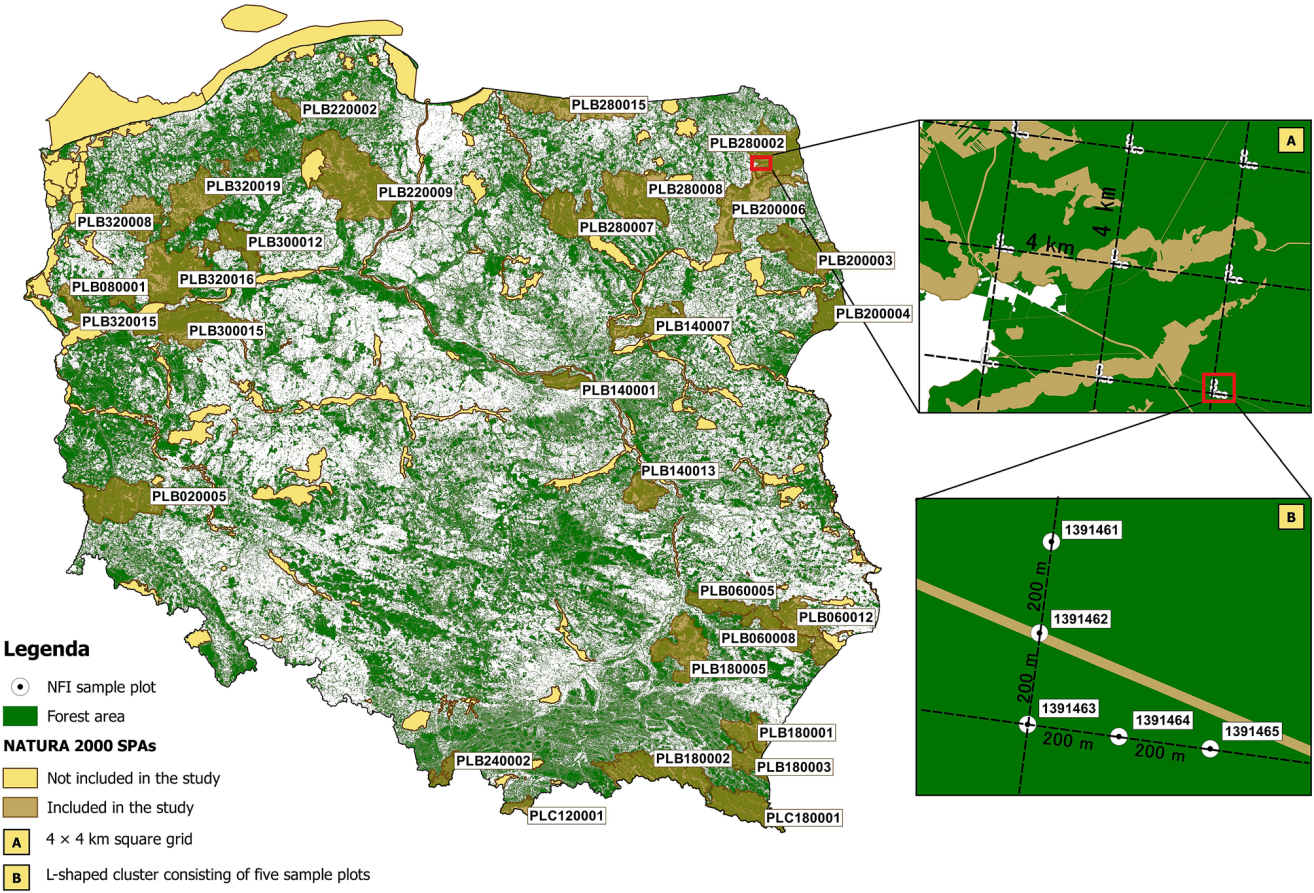
Special protection areas for birds included in the study and the arrangement of clusters of sample plots within the NFI grid (The map was generated based on data from the GDOS^45^ and BDL^46^ websites. The spatial data was then integrated in the Qgis 3.10^47^).

Poland has 145 SPAs with a total area of 4,911,399 ha (15.7% of the country’s overall territory). Most of them contain woodland areas including both high quality forest ecosystems (e.g., national parks and nature reserves) and managed forests, which constitute the vast majority of the afforested area of the country. In this study, the stands lying within SPAs were characterized on the basis of randomly placed sample plots monitored under the National Forest Inventory (NFI)^43^. All SPAs were analyzed as a whole (“SPA Poland”), and in addition 30 SPAs with the highest forest areas were described in greater detail (they are marked in Fig. 1 and characterized in Table 1). The 30 selected SPAs have an overall area of 3,215,625 ha, with individual SPAs ranging from 21,017 to 322,536 ha. SPAs reflect the entire range of natural conditions in Poland, from the lowlands to the Tatras (the highest mountains within the Carpathians). While the 30 SPAs contain a variety of natural habitat classes, most of them are dominated by forest habitat types such as broad-leaved deciduous woodland (N16), coniferous woodland (N17), and mixed woodland (N19)^44^. Furthermore, 16 of them exhibit a forest cover of more than 70%, with only five having less than 50% forest cover (Table 1).The dominant and co-dominant species in SPA stands (as determined on the sample plots) are *Pinus sylvestris*, *Picea abies*, *Fagus sylvatica*, *Quercus* sp*.*, *Betula pendula*, *Alnus glutinosa*, and *Abies alba*. SPAs constitute significant sites (refuges) for avian species such as the Eurasian three-toed woodpecker, Eurasian pygmy owl, and hazel grouse (Table 1, Supplementary Table S1 online). In addition to the species mentioned in Table 1, SPAs provide habitats to numerous other organisms, including those relying on the presence of deadwood in the ecosystem^2^.

**Table 1:** Shares of natural habitat classes in the 30 selected Special Protection Areas.

No	Natura 2000 SPA		SPA area	NFI sample plots (The share of sample plots located in parts of SPAs subject to strict protection)	Natural habitat class^a^								Dominant tree species^b^	Birds^c^
	Name	Code	[ha]	[no.] (%)	N4, N8, N9, N22	N6	N7, N10	N12, N21	N23	N16	N17	N19		
					Share in overall SPA area (%)									
1	Beskid Niski	PLB180002	151,966.61	350 (2)	0.05	0.17	10.78	14.43	0.31	21	18.06	35.2	A.a, F.s	AQP SXU DEL
2	Beskid Żywiecki	PLB140002	34,988.81	77 (4)	0.18	–	7.44	12.96	0.41	5.75	47.19	26.07	P.a, F.s	TU PIT DEL
3	Bieszczady	PLC180001	111,519.44	299 (19)	3.68	0.42	6.47	2.46	0.08	43.75	12.16	30.98	F.s, A.a	CN PA ACH SXU DEL PIT PIC FP FA
4	Bory Dolnośląskie	PLB020005	172,093.39	433 (0)	7.02	0.51	2.75	8.52	1.21	2.72	55.18	22.09	P.s	MML HA AF GLP CE TU
5	Bory Tucholskie	PLB220009	322,535.90	691 (0)	–	2.51	6.78	23.65	0.69	0.78	62.57	3.02	P.s	CE
6	Dolina Słupi	PLB220002	37,471.84	79 (0)	–	2.78	5.11	20.38	0.46	7.85	49.35	14.07	P.s, F.s	AF BB
7	Góry Słonne	PLB180003	55,036.88	122 (0)	0.32	0.04	10.69	18.47	0.16	22.95	17.03	30.34	A.a, F.s, P.s	CN PA ACH SXU DEL PIT PIC FP FA
8	Lasy Janowskie	PLB060005	60,235.75	166 (0)	0.16	2.78	4.47	4.05	0.34	2.29	70.42	15.49	P.s	TU CN PA HA CE
9	Lasy Puszczy nad Drawą	PLB320016	190,279.05	368 (1)	–	2.91	5.54	26.69	0.41	6.96	50	7.49	P.s	PH MML HA BB PA FP
10	Ostoja Biebrzańska	PLB200006	148,509.33	123 (11)	–	0.13	47.02	17.2	0.43	15.71	14.09	5.42	P.s, A.g, B.p	AQC
11	Ostoja Drawska	PLB320019	15,3906.15	211 (0)	0.75	5.40	5.25	40.33	0.75	11	26.25	10.27	P.s, F.s, B.p	HA AQP MML BB
12	Ostoja Ińska	PLB320008	87,710.94	88 (5)	0.16	3.99	7.97	50.23	0.88	14.16	8.92	13.69	P.s, Q, F.s, A.g, P.a, B.p	HA AQP MML
13	Ostoja Kozienicka	PLB140013	68,301.20	116 (0)	–	0.36	7.94	34.96	3.61	5.38	32.33	15.42	P.s, Q	CN
14	Ostoja Warmińska	PLB280015	145,341.99	143 (0)	–	0.29	5.89	70	0.51	6.66	2.30	14.35	A.g, B.p, P.a, Q, P.s	CN AQP
15	Ostoja Witnicko-Dębniańska	PLB320015	46,993.07	86 (0)	–	1.23	2.65	27.73	0.59	12.5	47.5	7.80	P.s	HA MML
16	Pogórze Przemyskie	PLB180001	65,366.31	131 (2)	0.05	1.53	3.93	29.61	0.43	20.22	17.08	27.15	F.s, P.s, A.a	CN AQP SXU PIC DEL FP FA
17	Puszcza Augustowska	PLB200002	134,377.72	296 (0)	–	5.4	4.27	12.92	0.07	4.51	57.81	15.02	P.s, P.a	TU BON PA AQP AF DRM PIT DEL
18	Puszcza Barlinecka	PLB080001	26,505.63	86 (0)	–	3.83	2.37	2.25	0.01	25.31	57.88	8.35	P.s, F.s	BB HA MML AQP
19	Puszcza Biała	PLB140007	83,779.74	175 (0)	–	0.18	9.25	26.38	0.87	4.87	52.65	5.80	P.s	CE DRM
20	Puszcza Białowieska	PLC200004	63,147.58	186 (13)	0.26	–	4.03	2.3	0.23	43.42	32.39	17.37	P.a, P.s, A.g, Q	DEM PIT DEL FA PA AQP BON GLP
21	Puszcza Kampinoska	PLC140001	37,640.49	88 (16)	–	–	13.82	9.72	0.61	20.30	45.47	10.08	P.s, A.g	CN
22	Puszcza Knyszyńska	PLB200003	139,590.23	321 (0)	–	0.05	11.35	14.91	0.22	7.09	45.56	20.82	P.s, P.a	BON PIT AF AQP PA FP DRM
23	Puszcza nad Gwdą	PLB300012	77,678.90	207 (2)	–	3.05	2.09	5.06	0.05	5.22	80.1	4.43	P.s	BC DRM CE HA BB AF
24	Puszcza Napiwodzko-Ramucka	PLB280007	116,604.69	291 (0)	–	6.01	7.64	10.44	0.62	4.08	63.77	7.44	P.s	PH HA AQP DEM
25	Puszcza Notecka	PLB300015	178,255.76	443 (0)	–	2.60	4.16	16.78	0.29	2.37	67.13	6.67	P.s	CE DRM HA MML MMG
26	Puszcza Piska	PLB280008	172,802.21	306 (0)	–	12.38	8.47	17.39	0.41	4.53	41.8	15.02	P.s	HA AQP AF
27	Puszcza Sandomierska	PLB180005	129,115.59	189 (0)	2.27	1.09	10.02	36.21	3.15	3.25	26.56	17.45	P.s	COG CN CE PIC
28	Puszcza Solska	PLB060008	79,349.09	204 (0)	–	0.52	8.51	12.26	0.76	1.70	71.19	5.06	P.s	CN PA HA AQP TU BB SXU AF CE DRM
29	Roztocze	PLB060012	103,503.33	156 (3)	–	0.48	4.52	40.65	1.48	5.33	31.73	15.81	P.s, F.s	CN PA AQP SXU PIC DRM DEL FP FA
30	Tatry	PLC120001	21,017.80	43 (47)	35.94	0.52	0.35	0.18	0.06	0.12	59.02	3.81	P.a, A.a	TU GLP AF PIT
Remaining 77 SPAs			1,450,849.09	1500										
Total			4,666,474.51	7974										

^a^According to the Standard Data Form^50^: N4—sand beaches; N6—inland water bodies (standing water, running water); N7—bogs, marshes, water fringed vegetation, fens; N8—heath, scrub, maquis and garrigue, phrygana; N9—dry grassland, steppes; N10—humid grassland, mesophile grassland; N12—extensive cereal cultures (including rotation cultures with regular fallowing); N16—broad-leaved deciduous woodland; N17—coniferous woodland; N19—mixed woodland; N21—non-forest areas cultivated with woody plants (including orchards, groves, vineyards, dehesas); N22—inland rocks, screes, N23—other land (including towns, villages, roads, waste places, mines, industrial sites);

^b^Tree species with a share of > 10% in overall stand volume (in descending order): A.a—*Abies alba*; A.g—*Alnus glutinosa*; B.p—*Betula pendula*; F.s—*Fagus sylvatica*; P.a—*Picea abies*; P.s—*Pinus sylvestris*; Q—*Quercus* sp.

^c^The table includes bird species listed in Annex I to the Birds Directive associated with large trees, primary and secondary cavity nesters, and deadwood associates, for which the analyzed SPAs are important habitats in Poland^2^: ACH: *Aquila chrysaetos*, AF: *Aegolius funereus*, AQC: *Aquila clanga*, AQP: *Aquila pomarina*, BB: *Bubo bubo*, BON: *Bonasa bonasia*, CE: *Caprimulgus europaeus*, COG: *Coracias garrulus*, CN: *Ciconia nigra*, DEL: *Dendrocopos leucotos*, DEM: *Dendrocopos medius*, DRM: *Dryocopus martius*, FA: *Ficedula albicollis*, FP: *Ficedula parva*, GLP: *Glaucidium passerinum*, HA: *Haliaeetus albicilla*, MMG: *Milvus migrans*, MML: *Milvus milvus*, PA: *Pernis apivorus*, PH: *Pandion haliaetus*, PIC: *Picus canus*, PIT: *Picoides tridactylus*, SXU: *Strix uralensis*, TU: *Tetrao urogallus.*

More detailed information about selected bird species found in the SPAs, including their densities, is provided in Table S1 in the supplementary materials (available online) based on data from the book by Wilk et al.^2^. The quantitative data given there include abundance figures obtained by different methods ranging from comprehensive inventories encompassing entire SPAs to approximate estimations based on fragmentary field observations. Fifty-one percent of the data was from exact counts, 10% from extrapolation (an exact inventory of part of an SPA, with the rest estimated based on the areas of different habitat types), and 17% from approximate estimates (no inventory, based on fragmentary field observations); no information about accuracy was available for the remaining 22% of the data. This dataset was sufficient for a preliminary determination of the significance of individual SPAs in terms of the protection of bird species. However, it should be noted that the provided estimates have an unknown measurement error, and so they should be used cautiously. They may also be inappropriate for some analyses, such as abundance change over time^2^.

To identify NFI sample plots located in SPAs, it was necessary to compare a layer containing the coordinates of NFI sample plots with a layer specifying the boundaries of all SPAs. The network of sample plots established in Poland for the purpose of NFI monitoring amounts to nearly 30,000 circular sample plots distributed on a 4 × 4 km grid. At every node of the grid, there is an L-shaped cluster of 5 sample plots spaced out at 200 m intervals (Fig. 1). Spatial analysis showed that 107 out of a total of 145 SPAs contain at least one NFI sample plot. The overall number of sample plots located in those 107 SPAs was 7974. Sample plots were assigned to SPAs based on comprehensive analysis of spatial data in ESRI Shapefile format: vector data on NFI sample plot location, vector and descriptive data on nature protection plans taken from CRFOP^44^, and vector data on forest areas from https://www.bdl.lasy.gov.pl/portal/. The source data were integrated in Qgis 3.10 software^47^ to generate a layer showing all sample plots located within the boundaries of individual SPAs. In the next step, 30 of the original 107 SPAs with sample plots were selected for further in-depth analysis (Fig. 1). This group consisted of 29 SPAs with the greatest number of NFI sample plots as well as the Tatry SPA, which was added to ensure that all terrain types found in SPA Poland are represented. The 30 selected SPAs contained from 43 to 691 sample plots each, a total of 6474 sample plots (Table 1).

Tree stand parameters were recorded by the NFI in the years 2010–2014^42^. Measurement data and descriptions of sample plot characteristics were made available to the authors by the State Forests National Forest Holding. Measurements were done in accordance with the methodology (approved by the Polish Ministry of Environment) set forth in the following publications: ME^48^, NFI^43^, and Talarczyk^49^. Individual sample plots had an area of 0.02–0.05 ha each. The monitored parameters included the density of living trees and shrubs as well as standing dead trees with a DBH of ≥ 7 cm. Living trees and standing entire dead trees had their height measured and DBH taken at 1.30 m above ground.

The height of snags was recorded and their diameter was either measured or estimated halfway their height. DBH and length were determined for entire downed trees, while other downed deadwood fragments had their diameter measured halfway their length (only fragments with a diameter of at least 10 cm at the thicker end were included). Deadwood decay was evaluated on a 3-point scale: stage I—non-decayed (unaltered wood structure, natural light color, no fungal or bryophyte growth), stage II—partially decayed (altered dark color of the wood, fungi or bryophytes present, rot), and III—strongly decayed (extensive fungal, lichen, and bryophyte growth, sometimes completely decomposed sapwood and partially preserved heartwood). Assessment also involved percentage seedling cover (trees and shrubs with a height of < 0.5 m), percentage sapling cover (trees and shrubs with a height of ≥ 0.5 m and DBH < 7 cm), and ground vegetation type. Every sample plot was evaluated based on habitat studies or vegetation assessment in terms of site conditions, that is, site fertility (dystrophic, oligotrophic, mesotrophic, eutrophic) and moisture (dry, mesic, moist, boggy). In addition, the plots were characterized in terms of the presence or absence of strict protection based on official documentation^43,48,49^.

### Data analysis

Analysis involved data from all 7974 sample plots located in Polish SPAs (a total of 107 SPAs collectively referred to as “SPA Poland”). Separate analysis was conducted for data from the 30 selected SPAs (Table 1). The focus was on the stand attributes important for birds associated with woodland areas^51–53^, that is, standing trees (and especially large ones), standing and downed deadwood, overall deadwood volume, and the general conditions found in the bottom forest layer, being a function of all components of that layer.

#### Standing deadwood and living trees

NFI data have certain limitations and require some fine distinctions. In this work, standing deadwood was defined as all standing dead elements with a height of at least 0.4 m (excluding post-harvest stumps up to 0.3 m). Data were used to calculate the volume and density of all those elements. The volume of snags was calculated from Huber’s formula. Tariff tables were used to determine the volume of entire standing trees^54^. The diameter of snags was measured halfway their height and their DBH was estimated adopting a mean taper of 1 cm/1 m. The relationship between DBH and height was calculated for standing entire dead trees and snags. Separate analysis was performed for large standing deadwood with DBH ≥ 30 cm. Similar calculations were done for living trees.

#### Downed deadwood and the space filling index

**Figure 2:**
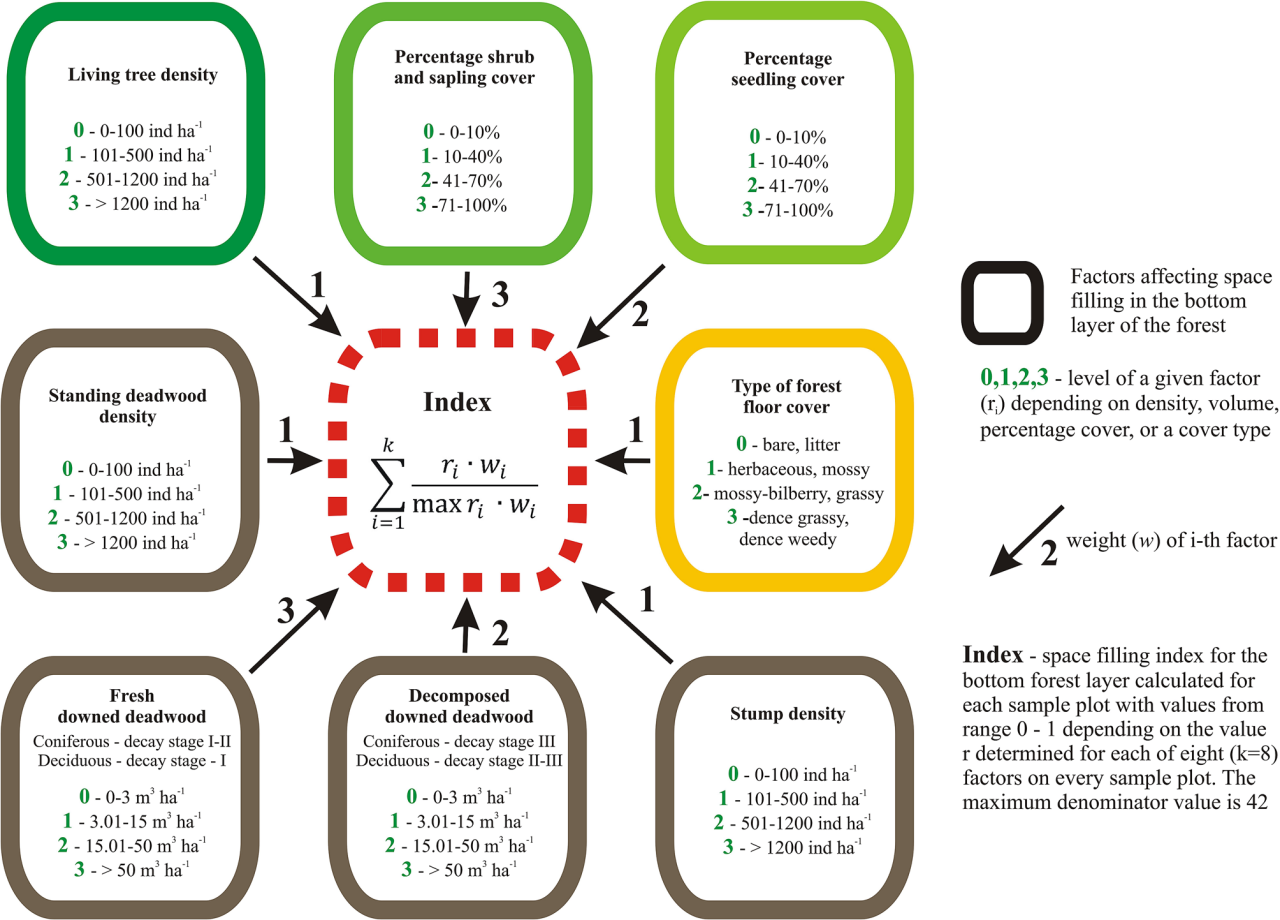
Calculation of the space filling index (SFI) for the bottom layers of the forest.

The volume of downed deadwood was calculated in the same way as that of standing deadwood. Downed deadwood was analyzed separately as well as in conjunction with other elements present in the bottom layers of the forest to provide a comprehensive picture of the habitat conditions in that layer, which may be conceptualized as the degree of space filling. The authors proposed an aggregate space filling index (SFI), having previously conducted a study involving a very similar indicator (combining various characteristics of the forest floor) for sites occupied by the Eurasian wren^55^. In order to bring the indicator in line with NFI methodology and present it on several clear examples (low or no space filling, medium filling, high filling, very high filling) additional measurements were performed (see Supplementary Fig. S1 online). In the present work, the SFI encompassed the trunks of standing living and dead trees, seedlings, saplings, ground vegetation, low post-harvest stumps, decayed downed deadwood, and fresh downed deadwood (Fig. 2). Each of the constituent elements was rated on a four-point scale with 0 denoting its absence or very low level, 1—low level, 2—high level, and 3—very high level. The criteria for this classification were developed from the authors’ experience. In addition, each element was given a weight according to its contribution to filling the space occupied by birds, from 1 (low contribution) to 3 (high contribution, e.g., saplings or fresh downed coniferous deadwood). The rating for each element in each sample plot was multiplied by its weight, with the sum of products reflecting the habitat conditions for a given plot (the higher the SFI, the greater the degree of space filling). The SFI may theoretically range from zero, which would mean the absence of any vegetation and/or deadwood, to 1, meaning a complete filling of the available space by all studied elements of vegetation. In practice an SFI of 1 cannot be reached either in natural forests or managed stands because the studied characteristics do not normally occur with the highest intensity at the same time. When calculating the SFI, the denominator is always 42, while the numerator rarely takes an overall score of more than 12 (see Fig. 2) In addition, field observations also show that the value of the numerator above 12 already reflects very high and diverse filling of the bottom forest layers (see Supplementary Fig. S1 online). For example, reaching a very high degree of space filling would require a 100% shrub and sapling cover or large amounts of fresh coniferous downed deadwood with some additional factors contributing the remaining 3 points. The results are presented as a distribution of sample plots between the four adopted levels of space filling in the bottom forest layers: a low degree of space filling or no filling (SFI < 0.10), a medium degree (0.10 ≤ SFI < 0.20), a high degree (0.20 ≤ SFI < 0.30), and a very high degree (SFI > 0.30) (see Supplementary Fig. S1 online).

#### The density of primary and secondary cavity nesters in SPAs

SPA location and site conditions affect the species composition of stands. This is significant in analyzing the occurrence of individual bird species with different geographic ranges and preferences in terms of stand species composition. Given the above and taking into consideration the characteristics of the data provided by Wilk et al.^2^, the present study analyzed a broad group of birds with similar ecological requirements, but associated with different stand types—primary and secondary cavity nesters. Importantly, data for their abundances in the analyzed SPAs were relatively complete. The density of primary and secondary cavity nesters listed in Annex I to the Birds Directive (a total of twelve species marked in Table S1) was calculated based on information from Wilk et al.^2^. If the authors specified a range of densities, the middle value was selected for the present study. Subsequently, the overall number of bird pairs belonging to all twelve species was computed for each SPA and divided by its forest area (forest areas were defined as sites with codes N16, N17, and N19, see Table 1). In this way, the overall density of primary and secondary cavity nesters was obtained for each of the 30 analyzed SPAs, in pairs per 1 km^2^ of forest area.

#### Statistical analysis

Multiple regression models were used to identify the factors affecting deadwood volume (model I) and the density of large standing deadwood (model II). The models were constructed on the basis of data from the 30 selected SPAs. Each SPA was described by several independent variables, including its location and sample plot attributes. The number of sample plots in each SPA is given in Table 1. Consequently, in this work, SPAs were used as sampling units for which the following variables were calculated based on data from the sample plots found in them:

- SPA location in the mountains [0–1]—a dichotomous variable (mountainous areas are defined as terrain with an elevation of more than 300 m with respect to the surrounding level, resulting from orogeny and volcanic activity) (the variable was used in models I and II);
- share of sample plots under strict protection within a given SPA[%] (harvest activities in a given area prohibited by law; the duration of strict protection varies according to the date of establishment of a given national park or nature reserve) (models I and II);
- share of sample plots located on mesotrophic and eutrophic sites (as opposed to oligotrophic and dystrophic ones) within a given SPA [%] (models I and II);
- share of sample plots located on moist and boggy sites (as opposed to mesic and dry ones) within a given SPA [%] (models I and II);
- mean volume of living trees [m^3^ ha^−1^] (models I and II);
- mean density of living trees [trees ha^−1^] (models I and II);
- mean density of living trees with DBH ≥ 30 cm [trees ha^−1^] (only in model II).

The models were built by the stepwise forward method. The normal distribution of residuals and homoscedasticity of variances were evaluated using the Kolmogorov–Smirnov test and the White test, respectively. The stability of the model was evaluated with the Chow test.

An additional analysis involved the relationship between deadwood volume and the density of primary and secondary cavity nesters in individual SPAs. Calculations were implemented in Statistica 13 software^56^.

## Results

### Total deadwood

**Figure 3:**
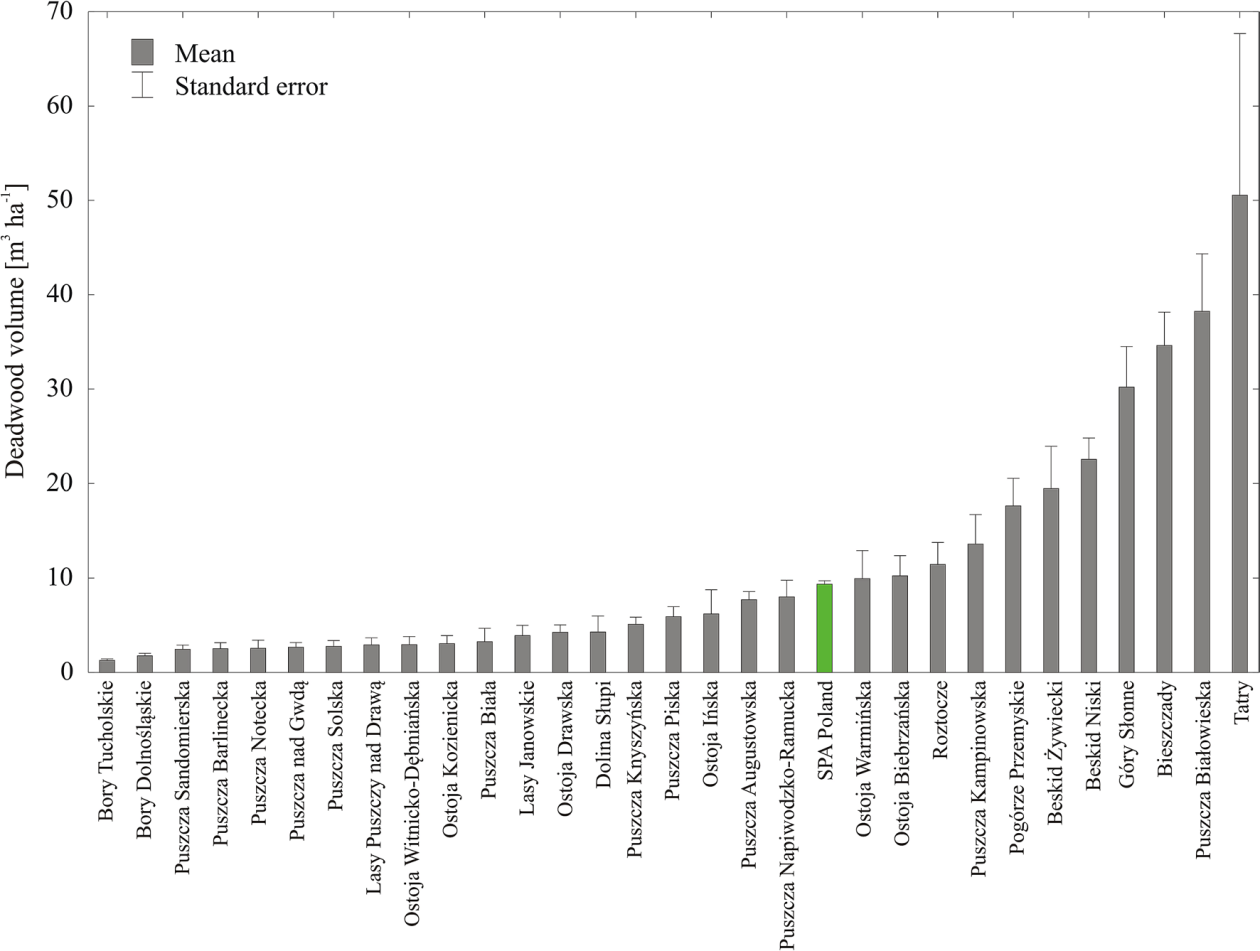
Mean deadwood volume in forests within the boundaries of SPA Poland and the 30 selected SPAs.

**Figure 4:**
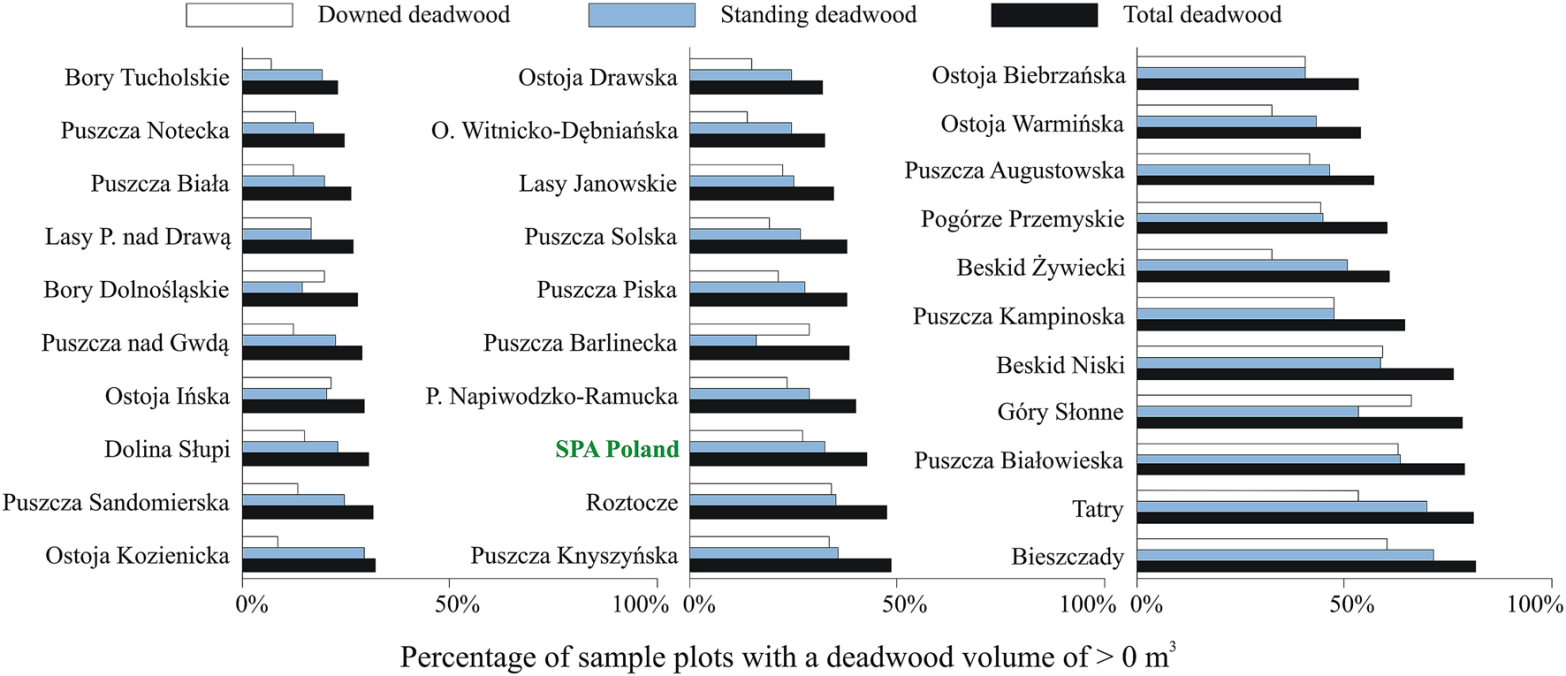
Percentage of sample plots in individual SPAs with a volume of downed, standing, and total (downed or standing) deadwood of > 0 m^3^.

Mean deadwood volume calculated for all sample plots located in 107 SPAs (“SPA Poland”) amounted to 9.4 m^3^ ha^−1^ (standard error SE = 0.37 m^3^ ha^−1^). In the 30 selected SPAs, deadwood volume ranged from 1.3 m^3^ ha^−1^ (0.2) to 50.5 m^3^ ha^−1^ (17.1), with a mean of 9.0 m^3^ ha^−1^ (0.4) (Fig. 3). On average, downed deadwood was present on 27%, and standing deadwood on 33%, of sample plots; either kind of deadwood was found on 43% of them. In individual SPAs, deadwood was found on 23–82% of sample plots (Fig. 4). In most SPAs, the sample plot frequency of standing deadwood was higher than that of downed deadwood. The species composition of deadwood varied considerably between the SPAs due to differences in site conditions (see Supplementary Fig. S2 online).

### Standing deadwood

Taking into consideration combined data for all SPAs, downed and standing deadwood accounted for 51% and 49% of the total deadwood volume, respectively (Supplementary Table S2 online). However, individual SPAs revealed considerable differences, with the share of standing deadwood ranging from 35 to 86%. The mean standing deadwood density was 30 ind ha^−1^ (SE = 0.8 ind ha^−1^), with the lowest value in individual SPAs being 8 ind ha^−1^ (SE = 2.4 ind ha^−1^), and the highest being 119 ind ha^−1^ (SE = 28.5 ind ha^−1^). The mean DBH of standing deadwood was 17.1 cm (median = 13.1 cm). In individual SPAs, the difference in mean DBH between sample plots was up to 13 cm, from 10.6 cm (median = 8.5 cm) to 23.1 cm (median = 18.0 cm). As much as 48% of standing deadwood was in decay stage II, with the proportion of fresh deadwood (decay stage I) accounting for 30%. The share of strongly decomposed standing deadwood was the lowest due to compromised trunk stability. However, those proportions varied greatly between SPAs (Supplementary Table S2 online).

**Figure 5:**
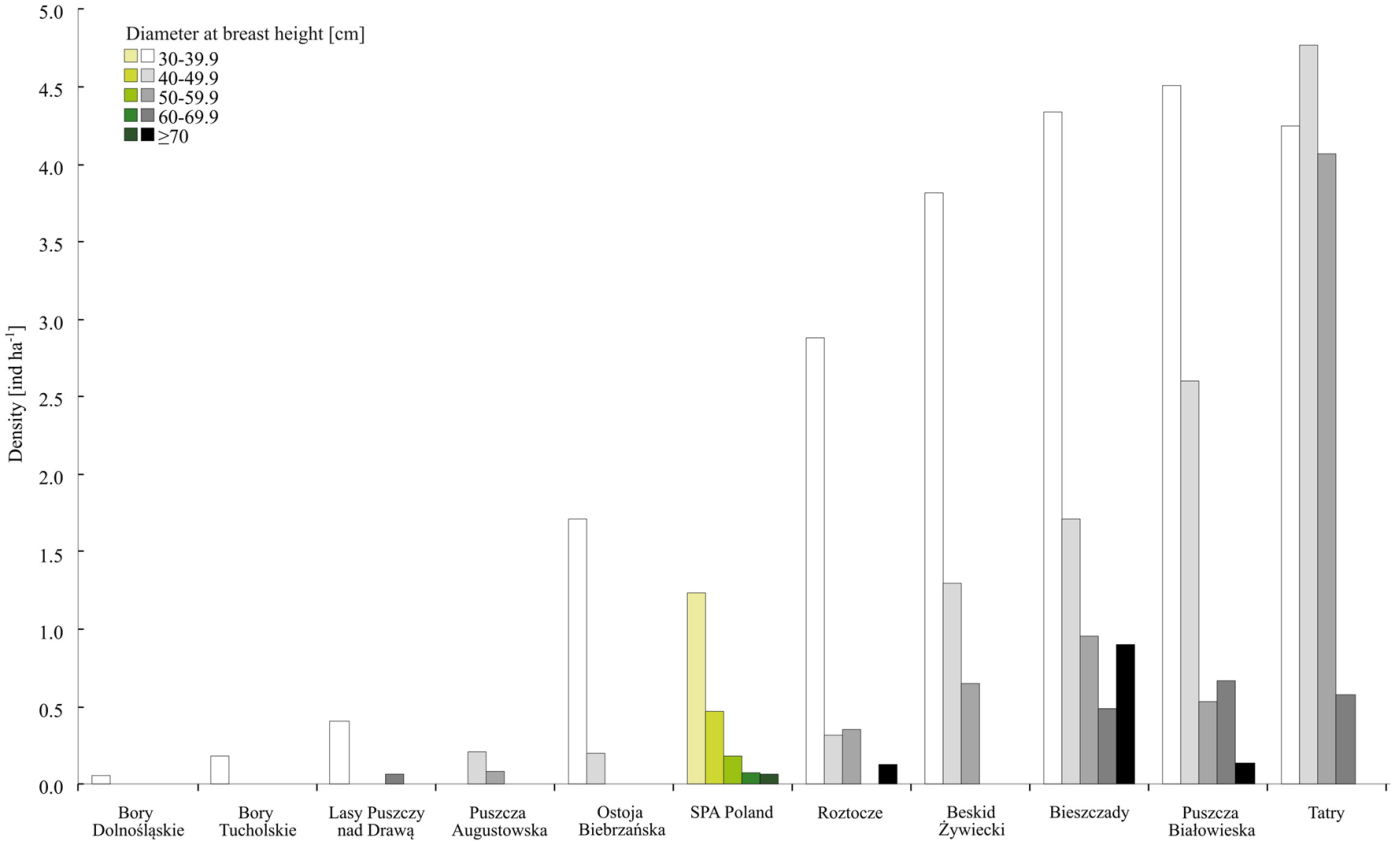
Structure of standing deadwood with DBH ≥ 30 cm for SPA Poland and 10 of the selected SPAs.

The mean density of large standing deadwood with DBH ≥ 30 cm was 2.2 ind ha^−1^ (SE = 0.1 ind ha^−1^, see Fig. 5). While some SPAs were almost completely devoid of such deadwood, with a density of 0.1 ind ha^−1^, others considerably exceeded the mean, with a density reaching 16.0 ind ha^−1^. Those values are relatively low as compared to the mean density of living trees with those dimensions (111 ind ha^−1^, SE = 1.2 ind ha^−1^, see Supplementary Table S2 online).

**Figure 6:**
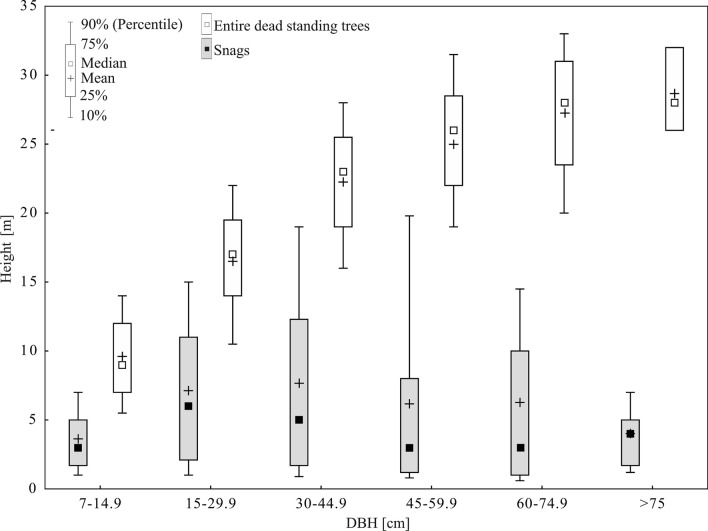
Relationship between the DBH and height of snags and entire dead standing trees (data for all 107 SPAs).

The relationship between the DBH and height of entire standing dead trees had the form of a height curve (Fig. 6). However, due to the rich species composition and differences in tree spacing, the ranges of tree height within the various DBH classes were wide. On the other hand, many snags with large DBH were short, and so the mean height was the greatest for DBH 30–44.9 cm, and decreased for greater DBH values (Fig. 6).

### Downed deadwood and the space filling index

The density of downed deadwood of all diameters and lengths was on average 24 ind ha^−1^ (SE = 0.7 ind ha^−1^, see Supplementary Table S3 online), ranging from 4 ind ha^−1^ (SE = 0.7) to 83 ind ha^−1^ (SE = 8.6) in individual SPAs. More than half of downed deadwood (53%) was in decay stage III, with only 15% of fresh deadwood (decay stage I). However, those proportions highly varied between SPAs, as did the relative shares of coniferous and deciduous downed deadwood, due to site conditions. While coniferous deadwood was almost entirely absent (6.8%) from some SPAs, it was found to be the vastly predominant type (99.9%) of downed deadwood in others. Coniferous deadwood accounted for 47.2% of total downed deadwood for SPA Poland.

The mean percentage sapling cover was 22% (SE = 0.3%), ranging from 12 to 35% in individual SPAs. The seedling cover of SPAs was much lower, from 1 to 4%, with a mean of 2%. Finally, the mean density of stumps from harvesting was 320 ind ha^−1^, with a minimum of 156 ind ha^−1^ and a maximum of 454 ind ha^−1^ (Supplementary Table S3 online).

**Figure 7:**
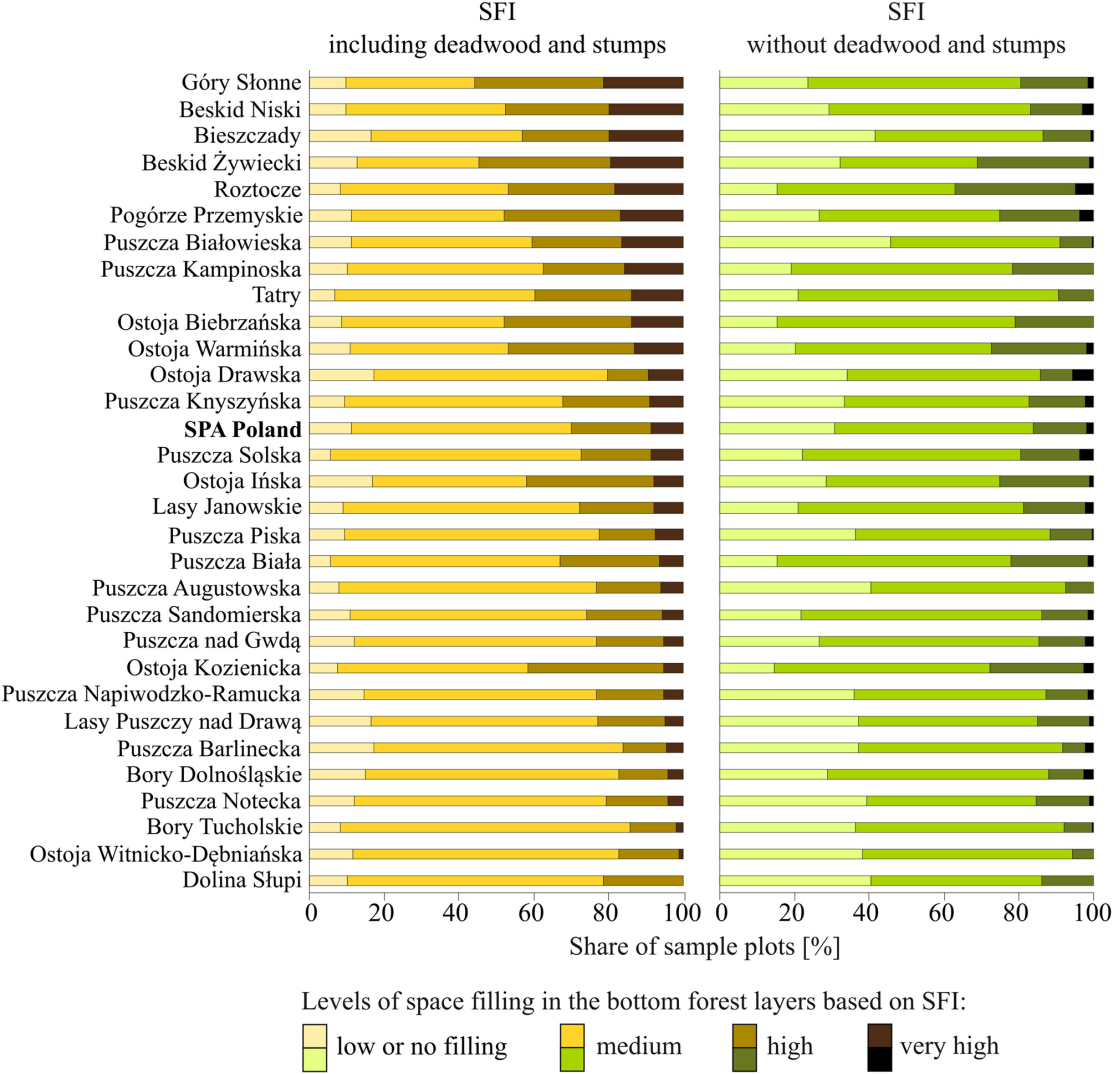
Variation in the space filling index (SFI) for the bottom layers of the forest.

We proposed an index that approximates conditions in the bottom forest layers. No such indicator is currently available in the literature. Taking into account all of the studied elements of the bottom forest layers, SFI values calculated for sample plots were from zero to 0.62. Space filling results obtained for only living trees, regeneration, and vegetation cover substantially diverged from SFI values in some SPAs, indicating a crucial role of deadwood in filling the bottom layers (Fig. 7). Extreme SFI values (the highest and lowest degrees of space filling) were rare, accounting for only 10% of the overall SPA area. Sample plots with a medium degree of space filling (0.10 ≤ SFI < 0.20) were the most numerous. Significant differences between SPAs were observed, with 6–18% of sample plots exhibiting a low degree of space filling, 32–77%—a medium degree, 11–36%—a high degree, and 0–21%, a very high degree. The percentage share of sample plots with high and very high SFI levels in individual SPAs ranged from 14 to 56% (Fig. 7).

### Factors affecting deadwood volume and the density of primary and secondary cavity nesters

The multiple regression models for deadwood volume (model I, Table 2) and the density of standing deadwood with DBH ≥ 30 cm (model II, Table 3) exhibited great similarity, with high determination coefficients R^2^ (0.86 and 0.87, respectively. The normal distribution of residuals and homoscedasticity of variances were satisfied in both models, *p* > 0.05 in the Kolmogorov–Smirnov and White tests. The stability of the model, *p* > 0.05 in the Chow test). Those parameters were significantly higher in SPAs with a greater number of sample plots under strict protection, a greater proportion of mesotrophic and eutrophic sites, and a higher mean stand volume (volume of living trees). In the case of model II, stand volume was strongly correlated (*R* = 0.88) with the density of living trees with DBH ≥ 30 cm. Nevertheless, stand volume led to a higher coefficient of determination in this model. In model I, a mountainous location also positively affected the value of the dependent variable. Neither model included site moisture or the mean density of living trees as the incorporation of those variables did not significantly improve regression.

**Table 2:** Linear regression results for deadwood volume.

Variable	Beta coefficient	Regression parameter	95% CI		*p*
Constant	–	− 10.095	− 24.479	4.289	0.161
Strict protection (%)	0.573	75.294	53.444	97.144	< 0.001
Mountainous location (0–1)	0.222	3.662	0.250	7.074	0.036
Volume of living trees (m^3^ ha^−1^)	0.202	0.053	0.007	0.100	0.026
Mesotrophic and eutrophic sites (%)	0.248	9.839	2.564	17.115	0.010

**Table 3:** Linear regression results for the density of standing deadwood with DBH ≥ 30 cm.

Variable	Beta coefficient	Regression parameter	95% CI		*p*
Constant	–	− 5.363	− 8.331	− 2.395	0.001
Strict protection (%)	0.668	22.217	17.181	27.253	< 0.001
Volume of living trees (m^3^ ha^−1^)	0.281	0.019	0.008	0.030	0.002
Mesotrophic and eutrophic sites (%)	0.298	2.994	1.310	4.678	0.001

**Figure 8:**
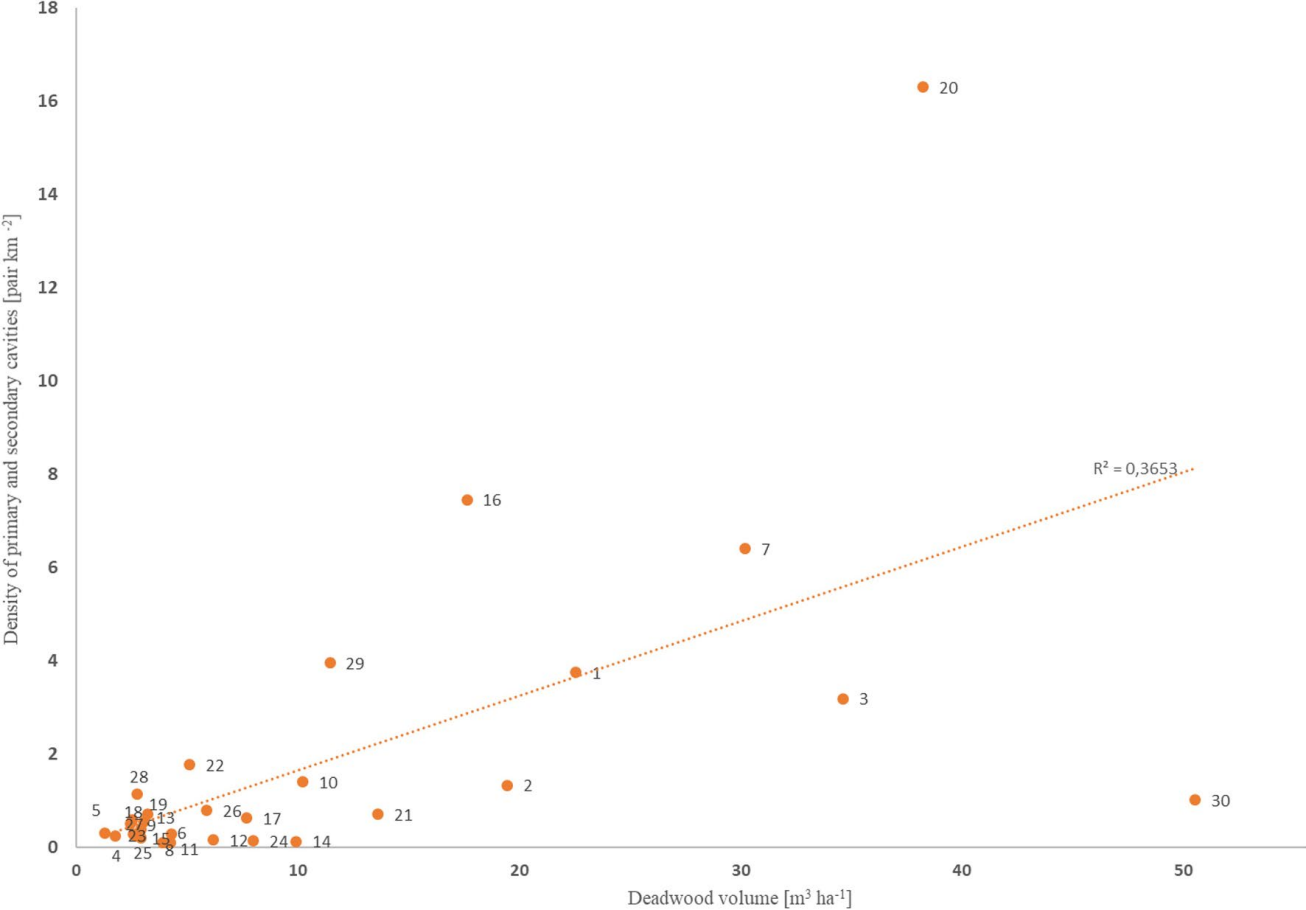
Relationship between the density of primary and secondary cavity nesters and deadwood volume in 30 SPAs. Analysis includes twelve bird species^2^ (see Table S1 in the supplementary materials as well as the “Study area and material” section). The numbers provided next to the points in the chart represent SPA numbers according to column 1 in Table 1.

The abundance of the analyzed primary and secondary cavity nesters was found to be generally higher in SPAs with greater deadwood volumes (*R* = 0.60, *p* < 0.05). By far the highest bird density was recorded in the Puszcza Białowieska SPA (no. 20, Fig. 8). In the Tatry SPA (no. 30, Fig. 8), the density of the analyzed bird group was relatively low despite a high deadwood volume. Much greater bird densities were found in areas with deadwood volumes exceeding 15–20 m^3^ ha^−1^ as compared to areas falling below that threshold.

## Discussion

Currently, the decline in animal populations may be primarily attributed to the loss of suitable habitats^57^, which has given rise to local and international conservation networks focused not only on the protection of animal species, but also on the preservation of their natural environments^58^. The success of conservation efforts depends on maintaining diverse woodland habitats both at the forest and landscape levels^35,59^. The Natura 2000 network covers the majority of Europe’s most biodiverse areas^60,61^. Comparative analyses of population trends provide strong evidence for positive associations between the degree of provision of certain conservation measures under the Birds Directive and the response of avian populations^62^. SPAs also fulfil an important role in bird migrations^63^. Nevertheless, the Natura 2000 network is not entirely free of deficiencies, and some authors have voiced the need for improvements in SPA functioning, including boundary adjustments and legal changes^64,65^. Natura 2000 sites are also being monitored to increase their effectiveness^3^. This work contains a review of SPAs in terms of the quality of forest habitats, with a direct bearing on the size and composition of avian assemblages^66,67^.

Very high differences between SPAs were found for deadwood volume (1.3–50.5 m^3^ ha^−1^). Deadwood volume is highly variable in woodlands across Europe, ranging from 5.6 to 33.1 m^3^ ha^−1^, with a mean of 15.8 m^3^ ha^−1^^68^, which is much greater than the mean for all Polish forests (5.9 m^3^ ha^−1^^43^), and also greater than the mean obtained in this study for SPA Poland (9.4 m^3^ ha^−1^). These differences are attributable not only to different management practices, but also to specific landscape characteristics, variation in site productivity, the occurrence of disturbances, and the proportion of protected areas^68^. Forests under similar protection plans may also differ in many respects as deadwood volume and composition are often largely determined by stand age, natural tree mortality, tree species composition, and terrain conditions^33^.

The present work revealed a positive relationship between the density of primary and secondary cavity nesters and deadwood volume in SPAs. However, the presented results should be interpreted cautiously due to certain limitations of the data on birds, which were characterized in detail in the data description^2^. There is no doubt that the identified relationship is real, but the results of future comprehensive bird inventories could modify its strength. Importantly, it should be remembered that the occurrence of secondary cavity nesters depends not only on the presence of the woodpeckers listed in Annex I to the Birds Directive, but also on the common woodpeckers, such as the Great spotted woodpecker *Dendrocopos major*. However, irrespective of their frequency, the presence of woodpeckers is determined by an abundance of deadwood-related microsites^69,70^. The density of cavity nesters was much higher in areas with a mean deadwood volume of over 15–20 m^3^ ha^−1^. While those threshold volumes are not very high, they are given for entire SPAs and they should be viewed in the context of the spatial distribution of dead trees. Deadwood is typically spread unevenly across forest ecosystems and so there are areas almost devoid of it as well as those very rich in deadwood, with volumes reaching hundreds of m^3^ ha^−1^^71^. The presence of areas with an accumulation of such microsites is decisive for many species^9^. At the same time, it should be noted that deadwood volume is but one of many parameters characterizing forest areas. The model developed for deadwood volume in SPAs indicates that it is linked to a number of other factors. Deadwood volume is also an indicator of the degree of SPA naturalness, which seems to be crucial in this relationship. Higher deadwood volumes not only improve ecosystem conditions for birds more or less associated with dead and dying trees, but also for many other organisms, and especially invertebrates. At the same time, a major difference was observed in the density of the analyzed bird group between two SPAs with high deadwood volumes, namely, Puszcza Białowieska and Tatry. The disparity may be explained by the site and climatic conditions prevalent in them, which largely determine the occurrence of bird species. Among the 30 analyzed SPAs, Tatry is the only SPA located in high mountains, which is reflected in the lower number of species and individuals found in those harsh conditions.

Within a given SPA, the factors beneficial for deadwood identified by the applied regression models include a higher percentage share of fertile sites and strict protection areas. Among the studied SPAs, those located in mountain and foothill regions reveal conditions particularly favorable for biodiversity. Due to a combination of multiple factors, the six SPAs with the highest deadwood volume include all analyzed mountain SPAs in the Carpathians. The abundance of deadwood is correlated with the richness of avifauna^40,69,70,72,73^. Although the Carpathians occupy only 6% of the overall area, they are home to 25% of the overall population of 24 out of 37 keystone bird species in this part of Europe, half of them being forest dwellers^74^. Taking into consideration their size and forest cover, Carpathian SPAs are much richer in species with international significance than lowland SPAs. Outside mountainous areas, deadwood is abundant in SPAs which encompass national park areas and in those where the proportion of spruce is similar to that in Carpathian SPAs. In general, the lowest deadwood volumes are found in those lowland SPAs which predominantly consist of managed forests. Deadwood levels in some of them are even lower than in managed forests in other countries^75^.

A substantial proportion of woodland bird species are to some degree associated with deadwood or niches arising from dying trees, such as gaps, herbaceous vegetation, dense regeneration, and other features. It has been reported that variation and changes in forest structure indicators alter proportions between canopy, cavity and ground/close to ground nesters^9^. SPAs differ both in terms of the density of large standing deadwood and the structure of the bottom forest layers. Mountainous SPAs exhibit a very high density of large standing dead trees (both entire trees and snags of various heights), which are critical both to primary and secondary cavity nesters, and especially the larger ones^76^. Mountainous SPAs are more abundant in standing deadwood, but it may be only a temporary phenomenon due to a periodic confluence of abiotic and biotic factors, such as those which have affected spruce stands over the past several years^77,78^. While windthrows may abruptly alter proportions between standing and downed deadwood, the short term bark beetle outbreaks increase the amount of standing large deadwood. As mountainous SPAs are not homogeneous in terms of their species composition, their resistance to disturbances may vary according to the properties of individual tree species. For instance, the beech, which is widespread in mountains, is characterized by relatively quick decomposition processes after dying^79^ and cannot remain standing for long due to mechanical reasons. As can be seen from this and other studies, the contribution of standing deadwood to total deadwood volume in general appears to be significantly higher in coniferous forests than in deciduous and mixed ones^33^.

It is a major challenge to adequately describe the diversity and density of woodland niches. To that end, this paper proposes a novel space filling index (SFI) for the bottom layers of the forest. Its variability reflects the degree of diversity in space filling on and near the forest floor. Disproportions in the filling of the bottom forest layers were found to be very high among SPAs. Many of them lacked diverse patches characterized by a high SFI, rich in shrubs, saplings, and deadwood, which are indispensable for supporting multiple bird species at the same time. Annex I to the Birds Directive lists species that require gaps (e.g., nightjar^80^), dense forest floor structures (e.g., hazel grouse;^81^), and both (e.g., capercaillie^82^). The key to the conservation of those species is to maintain diverse habitats on a large scale^83^. The SFI points to another important fact: on sites with little or no natural regeneration, deadwood offers the only opportunity to enhance forest structure, which is of exceptional significance for bird conservation. Indeed, were it not for deadwood, the bottom layer diversity of critical SPAs would not be very different from that of managed forests. Low bottom layer diversity is also characteristic of many Scots pine stands with a single story structure and with artificial regeneration planted in open areas or in gaps created by group selection practices. Thus, deadwood retention could substantially enrich and differentiate the bottom layers of those stands with great benefit for bird conservation.

In managed forests, the choice of felling systems and regeneration patterns is largely affected by site conditions. Short rotation periods may often limit the amount of standing deadwood, and especially of large dead trees, which play a central role in supporting biodiversity^84^. However, longer rotation periods would not necessarily solve the problem of deadwood structure as the density of large living trees is already quite high in all SPAs. However, while large trees are present in stands, they rarely remain in place until natural death and decomposition because the prevalent silvicultural paradigm does not encourage conscious efforts to make woodlands, including managed forests, more suitable for animal life^85^. In this context it should be emphasized that the structural enrichment of managed forests may be an effective method of increasing their attractiveness for deadwood-associated birds^86^ as large dead trees are required by woodpeckers (e.g., white-backed and black woodpeckers) as well as other species listed in Annex I to the Birds Directive, such as the red-breasted flycatcher, boreal owl, Ural owl, and others^74,87^. It could be beneficial to set aside areas within managed forests where timber harvesting is not profitable due to difficult terrain conditions (surface springs, steep slopes, ravines, and river banks). That would enable trees to grow to a large size and die naturally, making the structure of forests in SPAs more diverse. In lowland SPAs with more favorable terrain conditions, the improvement of habitat quality will require the setting aside of some forest areas for conservation purposes.

## Conclusions

The study results showed considerable differences between SPAs in terms of forest structural indicators. Very high variability among SPAs was found for deadwood volume and the density of large deadwood. As far as lowland managed forests are concerned, of particular importance is to reduce the removal of “economically useless elements,” including upturned root plates, from the bottom forest layer, and to increase the quantity of standing dead trees with DBH > 30 cm. In addition to measures aimed at the conservation of specific bird species, efforts should be made to improve the overall habitat conditions in those areas. For instance, one should consider the widespread adoption of retention forestry, including the exclusion of some old stands from timber production. In Scots pine forests characterized by a scarcity of saplings, downed deadwood can bring the much-needed diversity and improve low quality undifferentiated bird habitats. SPAs located in mountainous regions and those incorporating areas under strict protection tend to exhibit much better habitat conditions. In their case, conservation efforts may be geared toward the protection of individual bird species via monitoring and fine-tuning of the adopted forest management practices. Examples include birds which require specific habitat characteristics in addition to deadwood (e.g., the white-backed woodpecker needs old-growth beech stands abounding in large trees with dead branches). Special attention should also be paid to species requiring a combination of divergent habitats, for instance, gaps in forests in conjunction with patches of very dense vegetation providing food resources as well as cover for nests and chicks (e.g., capercaillie and hazel grouse).

## Supplementary Information

Supplementary Figure S1.

Supplementary Figure S2.

Supplementary Table S1.

Supplementary Table S2.

Supplementary Table S3.
