# Supplementary material for: Distribution of deadwood and other forest structural indicators relevant for bird conservation in Natura 2000 special protection areas in Poland
Source: Sci Rep. 2021 Jul 22;11:14937. doi: 10.1038/s41598-021-94392-1 (PMC8298385; doi:10.1038/s41598-021-94392-1)
Supplement: Supplementary file 1 — Supplementary Figure S1. [file 41598_2021_94392_MOESM1_ESM.docx]

The supplementary material describes the relationship between calculated SFI values and visual estimations of space filling in the bottom forest layers

The present work analyzes a vast number of sample plots located in a great variety of stands in both managed and unmanaged forests, reflecting the full range of space filling variability, from plots with no space filling to those with a maximum degree of filling. However, in the literature to date there is no indicator that would take into account all structural elements of the bottom forest layers, which provide habitats for numerous animal species, including birds.

Measurements from 58 sample plots were used to determine the relationship between the calculated SFI values and the visually estimated degrees of space filling in the bottom forest layers. The objective was to assign a range of SFI values to each visually estimated degree of space filling (Fig. S1). Each of the 58 sample plots had an area of 0.05 ha and measurements were conducted according to the methodology described in the manuscript. In addition, in the field each sample plot was visually classified into one of four degrees: no low or filling, medium filling, high filling, and very high filling. The choice of sample plots was subjective; the goal was to reflect the entire range of conditions in the analyzed stands (48 sample plots were located in the Gorce Mountains, five in the Świerklaniec Forest District, and five in the Jasień Nature Reserve). Among the 58 sample plots, 11 were visually classified in the low or no filling category, 18 in the medium filling category, 13 in the high filling category, and 16 in the very high filling category. Based on the relationship shown in Fig. S1, the following SFI ranges were adopted: <0.10 for low or no filling, 0.10≤SFI<0.20 for medium filling, 0.20≤SFI<0.30 for high filling, and SFI>0.30 for very high filling.

This approach to the determination of space filling in the bottom forest layers was previously analyzed by the present authors in a study that was aimed to correlate Eurasian wren locations with the structural conditions of the bottom forest layers (Oramus 2017). However, in the previous work, space filling was given as a sum of all the analyzed characteristics in contrast to the present paper, where it is expressed in the range from 0 to 1. Also the methodology of measuring space filling elements on sample plots has been slightly modified.


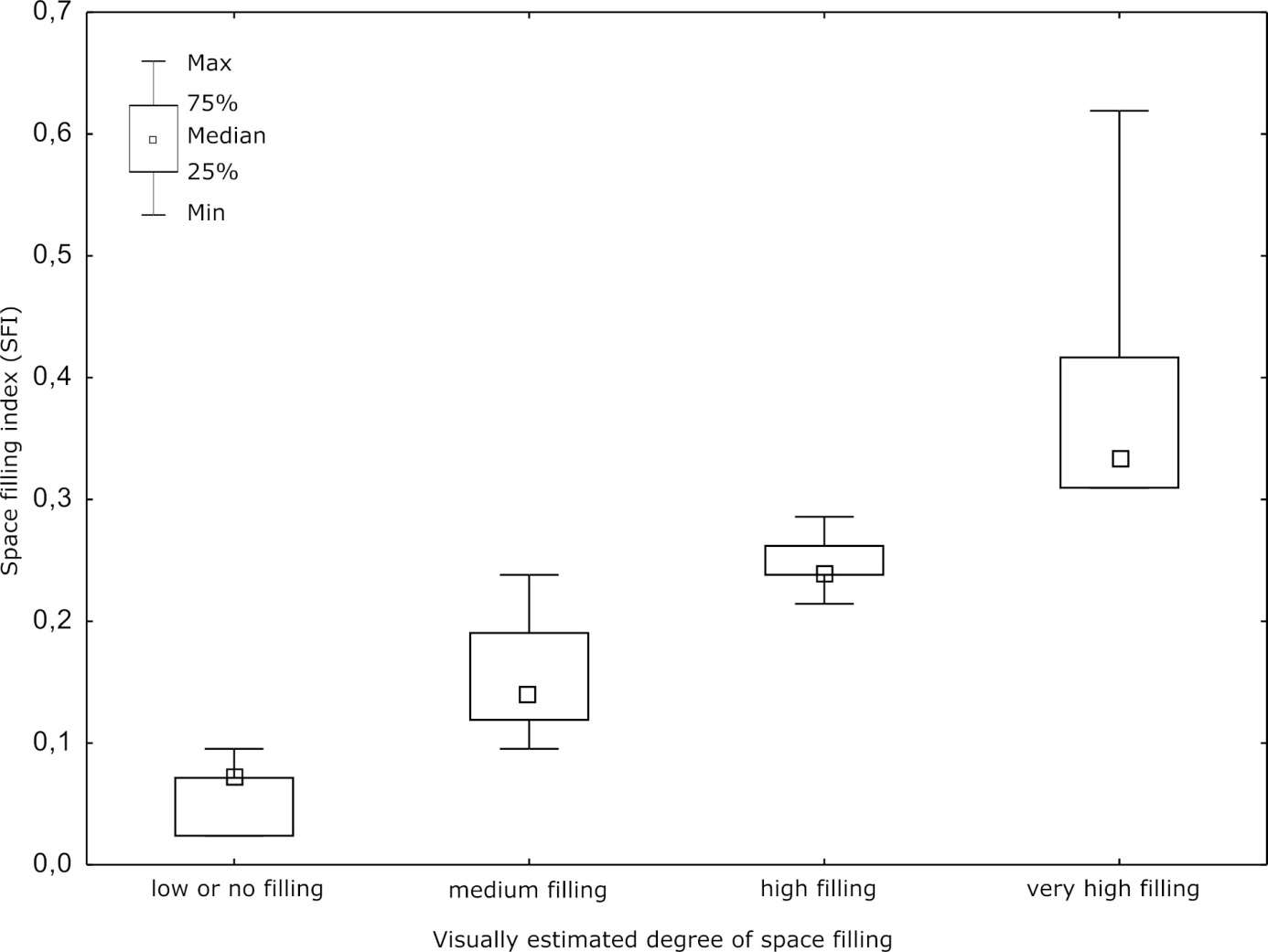


Fig. S1. Distribution of calculated SFI values among four degrees of space filling defined by visual estimation.
