# Supplementary material for: Distribution of deadwood and other forest structural indicators relevant for bird conservation in Natura 2000 special protection areas in Poland
Source: Sci Rep. 2021 Jul 22;11:14937. doi: 10.1038/s41598-021-94392-1 (PMC8298385; doi:10.1038/s41598-021-94392-1)
Supplement: Supplementary file 4 — Supplementary Table S2. [file 41598_2021_94392_MOESM4_ESM.docx]

Supplementary Table S2. Characteristics of standing deadwood and living trees

| SPA | Share of SDW in total DW volume | SDW volume by decay class – I/II/III | Density of living trees (SE) | Density of SDW (SE)^*^ | Mean DBH of SDW (median) | Density of living trees with DBH ≥30 cm (SE) | Density of SDW with DBH ≥30 cm (SE) |
| --- | --- | --- | --- | --- | --- | --- | --- |
|  | **%** | **%** | **ind ha^-1^** | **ind ha^-1^** | **cm** | **ind ha^-1^** | **ind ha^-1^** |
| **SPA Poland** | **49** | **30/48/22** | **733 (6.2)** | **30 (0.8)** | **17.1 (13.1)** | **111 (1.2)** | **2.2 (0.1)** |
| **SPA name** |  |  |  |  |  |  |  |
| Beskid Niski | 39 | 26/35/39 | 589 (19.9) | 59 (4.8) | 17.4 (12.6) | 144 (4.9) | 4.7 (0.9) |
| Beskid Żywiecki | 61 | 44/41/15 | 502 (49.0) | 50 (12.0) | 20.6 (17.8) | 141 (13.2) | 5.5 (1.6) |
| Bieszczady | 46 | 15/47/38 | 523 (22.5) | 89 (7.8) | 19.9 (14.2) | 127 (5.1) | 9.2 (1.3) |
| Bory Dolnośląskie | 52 | 17/71/12 | 822 (34.2) | 11 (1.8) | 13.6 (12.2) | 62 (4.0) | 0.1 (0.1) |
| Bory Tucholskie | 76 | 66/33/1 | 887 (23.8) | 12 (1.2) | 13.7 (11.8) | 91 (3.9) | 0.3 (0.1) |
| Dolina Słupi | 86 | 28/63/9 | 646 (61.4) | 17 (4.4) | 18.3 (15.9) | 123 (12.1) | 2.0 (1.4) |
| Góry Słonne | 43 | 24/60/16 | 444 (33.3) | 45 (6.1) | 23.1 (18.0) | 125 (7.6) | 7.3 (1.4) |
| Lasy Janowskie | 45 | 57/42/1 | 743 (37.2) | 15 (2.8) | 14.4 (11.0) | 128 (8.2) | 0.7 (0.4) |
| Lasy Puszczy nad Drawą | 50 | 22/63/15 | 856 (37.8) | 16 (2.9) | 14.1 (10.7) | 93 (5.9) | 0.5 (0.2) |
| Ostoja Biebrzańska | 45 | 12/69/19 | 750 (47.0) | 40 (5.6) | 16.3 (13.9) | 74 (8.6) | 2.1 (1.0) |
| Ostoja Drawska | 51 | 45/43/12 | 688 (34.8) | 19 (4.2) | 15.9 (13.5) | 132 (7.9) | 0.4 (0.3) |
| Ostoja Ińska | 35 | 14/71/15 | 570 (52.9) | 13 (3.3) | 18.6 (16.7) | 102 (12.3) | 1.1 (0.7) |
| Ostoja Kozienicka | 85 | 49/22/29 | 794 (48.6) | 18 (3.9) | 16.7 (14.0) | 122 (9.1) | 1.4 (0.6) |
| Ostoja Warmińska | 64 | 5/75/20 | 679 (38.7) | 44 (7.5) | 15.3 (13.5) | 76 (7.3) | 1.9 (1.2) |
| Ostoja Witnicko-Dębniańska | 76 | 76/23/1 | 763 (46.1) | 12 (2.6) | 17.1 (14.1) | 138 (12.9) | 0.3 (0.3) |
| Pogórze Przemyskie | 44 | 37/44/19 | 487 (27.2) | 34 (5.7) | 22.6 (17.8) | 146 (9.3) | 5.5 (1.3) |
| Puszcza Augustowska | 51 | 43/39/18 | 825 (28.5) | 48 (5.0) | 13.5 (11.4) | 119 (6.0) | 1.2 (0.4) |
| Puszcza Barlinecka | 37 | 1/51/48 | 748 (62.4) | 8 (2.4) | 16.6 (15.7) | 140 (12.4) | 1.2 (0.7) |
| Puszcza Biała | 53 | 16/79/5 | 736 (36.2) | 14 (3.0) | 10.6 (8.5) | 116 (8.7) | 0.5 (0.3) |
| Puszcza Białowieska | 43 | 27/44/29 | 786 (31.0) | 59 (5.9) | 20.5 (15.7) | 125 (7.0) | 10.6 (2.7) |
| Puszcza Kampinoska | 58 | 9/45/46 | 701 (48.6) | 75 (27.0) | 15.1 (12.2) | 120 (12.1) | 1.7 (0.8) |
| Puszcza Knyszyńska | 49 | 28/60/12 | 724 (25.5) | 29 (3.4) | 13.5 (10.5) | 117 (5.9) | 1.2 (0.4) |
| Puszcza nad Gwdą | 76 | 31/64/5 | 903 (52.3) | 17 (3.8) | 15.5 (13.0) | 93 (8.0) | 0.4 (0.3) |
| Puszcza Napiwodzko-Ramucka | 51 | 12/48/40 | 613 (27.8) | 21 (2.7) | 17.6 (14.8) | 143 (7.0) | 1.7 (0.5) |
| Puszcza Notecka | 56 | 27/65/8 | 942 (34.5) | 10 (1.4) | 16.7 (15.5) | 77 (4.6) | 0.4 (0.2) |
| Puszcza Piska | 54 | 31/48/21 | 621 (22.3) | 23 (3.1) | 14.6 (11.2) | 132 (6.2) | 1.4 (0.5) |
| Puszcza Sandomierska | 55 | 60/37/3 | 856 (44.4) | 21 (4.4) | 12.9 (10.8) | 93 (7.2) | 0.4 (0.2) |
| Puszcza Solska | 74 | 96/4/0 | 634 (32.1) | 15 (2.6) | 14.2 (11.3) | 112 (7.7) | 0.8 (0.4) |
| Roztocze | 62 | 82/13/5 | 659 (33.4) | 25 (4.2) | 19.9 (16.7) | 149 (9.0) | 3.5 (1.2) |
| Tatry | 62 | 15/82/3 | 719 (108.0) | 119 (28.5) | 16.6 (11.8) | 125 (20.3) | 16.0 (6.7) |

* – Density is given for all standing individuals (except stumps) irrespective of height; DW – deadwood, SDW – standing deadwood, SE – standard error.
