# Supplementary material for: Distribution of deadwood and other forest structural indicators relevant for bird conservation in Natura 2000 special protection areas in Poland
Source: Sci Rep. 2021 Jul 22;11:14937. doi: 10.1038/s41598-021-94392-1 (PMC8298385; doi:10.1038/s41598-021-94392-1)
Supplement: Supplementary file 5 — Supplementary Table S3. [file 41598_2021_94392_MOESM5_ESM.docx]

Supplementary Table S3. Characteristics of downed deadwood and lower forest layers

| SPA Poland | Downed deadwood density | Decay class share by deadwood volume – I/II/III | Coniferous species share by downed deadwood volume | Percentage seedling cover | Percentage sapling cover | Stump density | Dominant forest floor cover |
| --- | --- | --- | --- | --- | --- | --- | --- |
|  | ind ha-1 | % | % | % | % | ind ha-1 |  |
| **SPA Poland** | 24 (0.7) | 15/32/53 | 47.3 | 2 (0.1) | 22 (0.3) | 320 (3.2) | Grassy |
| **SPA name** |  |  |  |  |  |  |  |
|  |  |  |  |  |  |  |  |
| Beskid Niski | 61 (5.3) | 14/18/68 | 56.0 | 2 (0.3) | 26 (1.4) | 247 (11) | Litter |
| Beskid Żywiecki | 29 (7.3) | 56/27/17 | 86.7 | 4 (0.7) | 34 (3.3) | 437 (39) | Litter |
| Bieszczady | 61 (5.6) | 13/24/63 | 31.6 | 2 (0.3) | 20 (1.4) | 156 (10) | Herbaceous |
| Bory Dolnośląskie | 13 (1.9) | 18/34/48 | 71.2 | 2 (0.3) | 17 (1.1) | 316 (14) | Moss-bilberry |
| Bory Tucholskie | 4 (0.7) | 36/29/35 | 57.3 | 1 (0.1) | 12 (0.8) | 454 (13) | Mossy |
| Dolina Słupi | 4 (1.2) | 74/2/24 | 91.5 | 4 (1.4) | 17 (2.7) | 410 (31) | Grassy |
| Góry Słonne | 52 (5.5) | 18/22/60 | 59.2 | 1 (0.3) | 23 (2.2) | 203 (19) | Dense weedy |
| Lasy Janowskie | 11 (2.0) | 14/31/55 | 55.6 | 1 (0.2) | 26 (2.1) | 245 (18) | Grassy |
| Lasy Puszczy nad Drawą | 13 (2.9) | 5/50/45 | 30.9 | 3 (0.4) | 19 (1.4) | 351 (17) | Grassy |
| Ostoja Biebrzańska | 42 (6.2) | 8/43/49 | 16.9 | 1 (0.3) | 28 (2.3) | 231 (29) | Dense grassy |
| Ostoja Drawska | 10 (2.3) | 19/34/47 | 55.6 | 3 (0.5) | 20 (1.9) | 271 (19) | Grassy |
| Ostoja Ińska | 15 (4.1) | 0/25/75 | 16.3 | 3 (0.8) | 32 (3.5) | 245 (27) | Grassy |
| Ostoja Kozienicka | 5 (2.0) | 0/17/83 | 65.8 | 2 (0.5) | 35 (2.6) | 309 (21) | Litter |
| Ostoja Warmińska | 27 (4.5) | 8/29/63 | 10.8 | 2 (0.3) | 30 (2.4) | 309 (27) | Herbaceous |
| Ostoja Witnicko-Dębniańska | 8 (2.6) | 61/19/20 | 36.6 | 2 (0.4) | 15 (1.8) | 374 (30) | Grassy |
| Pogórze Przemyskie | 32 (5.2) | 26/34/40 | 62.2 | 3 (0.5) | 32 (2.6) | 236 (20) | Herbaceous |
| Puszcza Augustowska | 37 (3.9) | 9/25/66 | 40.8 | 1 (0.1) | 15 (1.1) | 318 (14) | Moss-bilberry |
| Puszcza Barlinecka | 22 (6.4) | 12/22/66 | 40.0 | 2 (0.4) | 16 (2.3) | 383 (38) | Litter |
| Puszcza Biała | 9 (2.8) | 2/5/93 | 6.8 | 2 (0.3) | 29 (2.1) | 364 (20) | Moss-bilberry |
| Puszcza Białowieska | 83 (8.6) | 9/36/56 | 46.9 | 3 (0.4) | 17 (1.6) | 260 (16) | Herbaceous |
| Puszcza Kampinoska | 50 (9.0) | 4/45/51 | 49.6 | 3 (0.6) | 26 (2.6) | 198 (22) | Moss-bilberry |
| Puszcza Knyszyńska | 29 (3.5) | 21/28/50 | 62.2 | 2 (0.3) | 25 (1.4) | 340 (14) | Herbaceous |
| Puszcza nad Gwdą | 7 (1.5) | 9/36/55 | 67.4 | 4 (0.9) | 18 (2.0) | 320 (20) | Grassy |
| Puszcza Napiwodzko-Ramucka | 18 (3.4) | 8/29/63 | 42.8 | 2 (0.2) | 19 (1.5) | 313 (16) | Grassy |
| Puszcza Notecka | 8 (1.6) | 20/57/23 | 33.2 | 1 (0.2) | 20 (1.3) | 316 (13) | Mossy |
| Puszcza Piska | 24 (7.1) | 12/28/60/ | 52.2 | 3 (0.4) | 19 (1.3) | 359 (17) | Moss-bilberry |
| Puszcza Sandomierska | 10 (2.7) | 42/34/24 | 55.9 | 1 (0.3) | 23 (1.7) | 323 (22) | Grassy |
| Puszcza Solska | 8 (1.6) | 35/45/20 | 72.3 | 2 (0.3) | 27 (2.0) | 272 (16) | Moss-bilberry |
| Roztocze | 22 (3.2) | 22/31/47 | 26.9 | 2 (0.4) | 29 (2.5) | 267 (16) | Herbaceous |
| Tatry | 42 (9.3) | 38/28/34 | 99.9 | 1 (0.2) | 15 (3.1) | 325 (47) | Grassy |

Types of forest floor covers: bare – no litter or ground vegetation; litter – litter without ground vegetation; herbaceous – patches of herbaceous plants; mossy – a moss carpet covers most of or the entire sample plot; moss-bilberry – moss carpets alternating with bilberry patches; grassy – patches of grasses with shallow root systems or bilberries; dense grassy – mostly a dense grassy cover with a matted root system – regeneration or afforestation requires agricultural treatment; dense weedy – dense ground vegetation consisting of plants with extensive and deep root systems or producing rhizomes or stolons preventing regeneration or afforestation without agricultural treatment.
